# Efficacy and safety of low-dose Sirolimus in Lymphangioleiomyomatosis

**DOI:** 10.1186/s13023-018-0946-8

**Published:** 2018-11-14

**Authors:** Hee-Young Yoon, Jung Jin Hwang, Dong Soon Kim, Jin Woo Song

**Affiliations:** 1 Departments of Pulmonary and Critical Care Medicine, Asan Medical Center, University of Ulsan College of Medicine, 88, Olympic-Ro 43-Gil, Songpa-Gu, Seoul, 05505 Republic of Korea; 2 Department of Convergence Medicine, Asan Medical Center, University of Ulsan College of Medicine, 88, Olympic-Ro 43-Gil, Songpa-Gu, Seoul, 05505 Republic of Korea

**Keywords:** Lymphangioleiomyomatosis, Sirolimus, Low dose, Respiratory function tests, Treatment outcome

## Abstract

**Background:**

Lymphangioleiomyomatosis is a rare disease caused by unregulated activation of mammalian target of rapamycin (mTOR) signalling pathway. Sirolimus showed efficacy in a phase 3 trial of patients with lymphangioleiomyomatosis, but the optimal dose remains unclear.

**Methods:**

We investigated the efficacy and safety of low-dose compared with conventional-dose sirolimus. Clinical data of 39 patients with lymphangioleiomyomatosis (mean age, 34.8 years; median treatment period, 29.6 months) who received sirolimus were retrospectively reviewed. Low-dose sirolimus was defined as any dose that maintained mean blood trough levels lower than those maintained with conventional doses (5–15 ng/mL).

**Results:**

Fifty-one percent of patients received low-dose therapy. The rate of decline in lung function decreased after treatment in the whole group (forced expiratory volume in 1 s [FEV_1_], − 0.12 ± 0.47 [before] vs. 0.24 ± 0.48% predicted/month [after], *p* = 0.027; diffusing capacity for carbon monoxide [DLco], − 0.33 ± 0.61 vs. 0.03 ± 0.26% predicted/month, *p* = 0.006) compared with before treatment. In the low-dose group, the rate of decline in FEV_1_ (− 0.08 ± 0.38 [before] vs. 0.19 ± 0.51% predicted/month [after], *p* = 0.264) and DLco (-0.13 ± 0.62 vs. 0.02 ± 0.28% predicted/month, *p* = 0.679) showed a numeric trend towards improvement after treatment; however, the conventional-dose group showed significant improvement in FEV_1_ (− 0.26 ± 0.54 [before] vs. 0.22 ± 0.38 [after] % predicted/month, *p* = 0.024) and DLco (− 0.55 ± 0.58 vs. 0.04 ± 0.25% predicted/month, *p* = 0.002) after treatment. Adverse events (AEs) occurred in 89.7% of patients and the most common AEs was hypercholesterolaemia (43.6%), followed by stomatitis (35.9%). The occurrences of AE were similar between the low- and conventional-dose groups (85.0% vs. 94.7%, *p* = 0.605).

**Conclusions:**

Low-dose sirolimus may stabilise lung function decline in lymphangioleiomyomatosis patients, but its efficacy appears to be inferior to that of conventional-dose sirolimus.

**Electronic supplementary material:**

The online version of this article (10.1186/s13023-018-0946-8) contains supplementary material, which is available to authorized users.

## Background

Lymphangioleiomyomatosis (LAM) is a rare progressive lung disease that mainly affects women of child-bearing age [1, 2]. The disease occurs sporadically or in association with tuberous sclerosis complex (TSC), and both are characterised by smooth muscle cell infiltration, cystic lung destruction, systemic angiomyolipoma (AML) and lymphangioleiomyoma formation [2, 3]. LAM is caused by mutations in the tuberous sclerosis genes, resulting in activation of the mammalian target of the rapamycin complex 1 (mTORC1) signalling network [4], a protein kinase that controls cell growth, proliferation and survival, and contributes to the uncontrolled proliferation of LAM cells [5]. Sirolimus, a highly specific inhibitor of mTORC1, can suppress the growth of spontaneously occurring renal tumours in a *Tsc2*^*+/−*^ Eker rat model [6] and in *Tsc1*^*+/−*^ and *Tsc2*^*+/−*^ mice [7], as well as in TSC2-deficient xenograft tumours in immune-deficient mice [8].

Based on early preclinical data, trials of sirolimus therapy in human tuberous sclerosis or LAM have been performed [9–11]. In a phase 3 trial of patients with LAM, sirolimus improved lung function, quality of life and functional performance [10]. In that study, the blood trough level of sirolimus was maintained between 5 and 15 ng/mL, based on a previous phase 1–2 trial [9]. However, the optimal treatment dose was not given because a significant number of patients developed problematic side effects, such as stomatitis, and the potential risk of developing a malignant tumour increased with long-term use [12]. In a recent study, low-dose sirolimus treatment (trough level < 5 ng/mL) was shown to improve lung function in nine patients without chylous effusion and to resolve chylothoraxes in seven patients with chylous effusions [13]. That study was limited by the small number of patients and the absence of comparison with conventional-dose therapy. Therefore, we aimed to compare the efficacy and safety of low- and conventional-dose sirolimus in patients with LAM.

## Materials and methods

### Study population

**Fig. 1:**
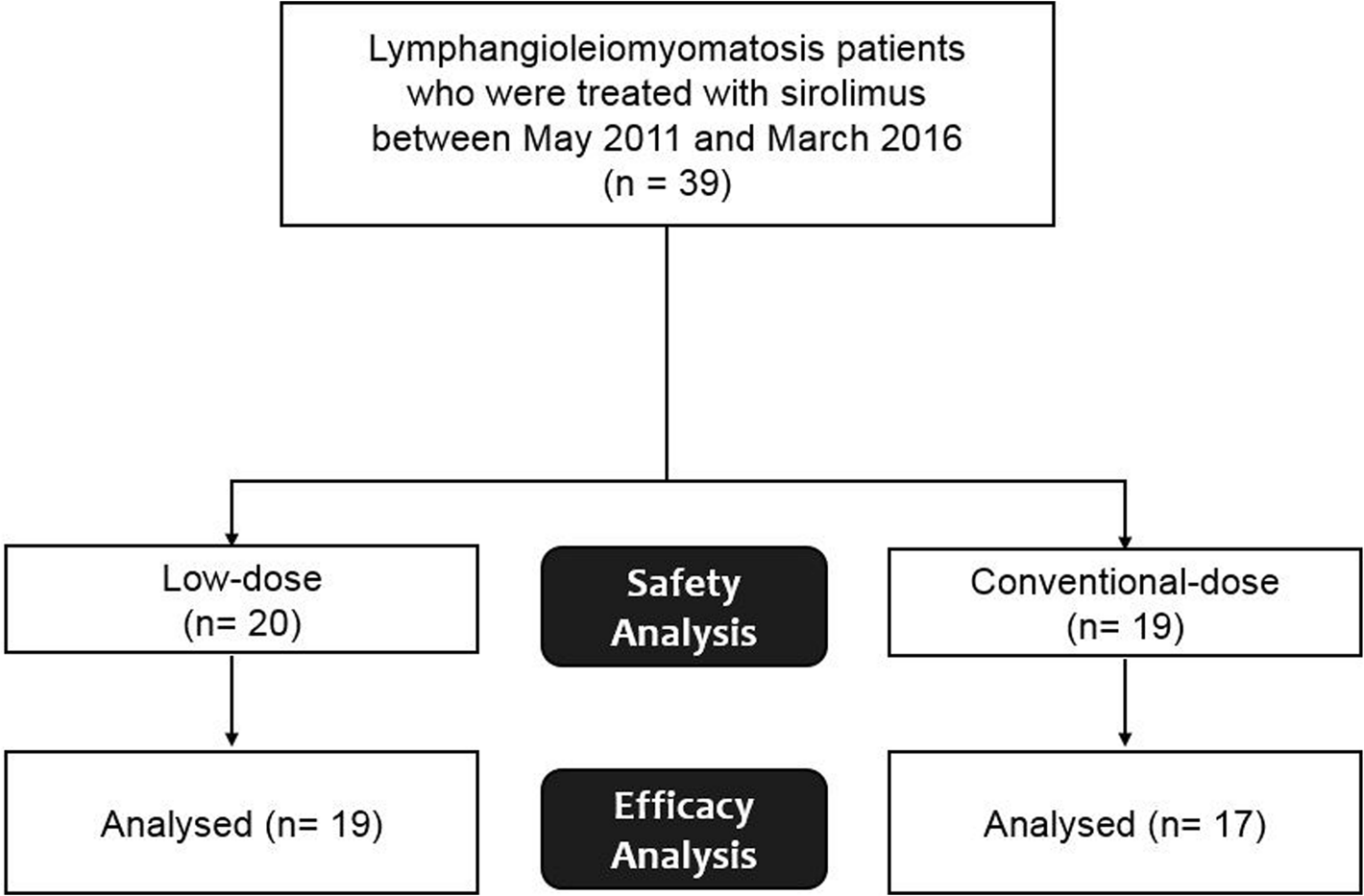
Flowchart of patients included from the analysis

The present study included 39 patients with LAM (82.1% biopsy-proven cases) treated with sirolimus between May 2011 and March 2016 at Asan Medical Center, Seoul, Republic of Korea (Fig. 1). All subjects met the diagnostic criteria of the American Thoracic Society/Japanese Respiratory Society guideline [14]. Subjects treated with sirolimus, who had mean blood trough levels maintained < 5 ng/mL, were classified as the low-dose sirolimus group. Informed consent was waived, and the study was approved by the Asan Medical Center Institutional Review Board (2016–0480).

### Methods

Clinical and survival data for all patients were retrospectively obtained from medical records, telephone interviews and/or National Health Insurance records. All subjects were routinely follow-up at 3-month intervals, and pulmonary function tests and measurement of blood sirolimus levels were performed at each follow-up visit. Whole blood sirolimus levels were measured by liquid chromatography-tandem mass spectrometry (LC-MS). Spirometry and measurement of the diffusing capacity of the lung for carbon monoxide (DLco) were performed according to the recommendations of the American Thoracic Society (ATS) and European Respiratory Society, and the results were expressed as percentages of normal predicted values [15–17]. The six-minute walk test was performed according to ATS guidelines [18].

### Evaluation of efficacy and safety

Efficacy was evaluated in patients who were treated with sirolimus for more than 12 weeks and who underwent pulmonary function tests more than three times before and after treatment (Fig. 1). Changes in lung function, specifically, forced expiratory volume in 1 s (FEV_1_), forced vital capacity (FVC) and DLco, from baseline to 12 or 24 months before and after treatment were evaluated. The rate of decline in lung function was estimated by linear regression modelling and compared before and after treatment. For categorical comparison, disease progression was defined as any decline in FEV_1_ during the observation period. The treatment response of extra-pulmonary manifestations was assessed in patients with AML or lymphangioma who had follow-up CT images using the Response Evaluation Criteria in Solid Tumours (RECIST) criteria (version 1.1) [19] and classified as follows: complete response (completely disappeared tumor), partial response (≥ 30% decrease in the sum of longest diameters of target lesions), progression (≥ 20% increase in the sum of longest diameters), and stability (all other changes). In this study, complete or partial responses were regarded as ‘improvement’.

Safety was evaluated in all patients who received at least one dose of sirolimus (Fig. 1). AEs were identified from the initiation of treatment to 28 days after the last dose and were classified using the preferred terms in the Common Terminology Criteria for Adverse Events (Version 4.0). SAEs were defined as any AEs occurring with any dose that resulted in any of the following outcomes: death, hospitalisation for life-threatening causes, disability or permanent damage, intervention to prevent permanent impairment or damage, or other serious medical events.

### Statistical analysis

All values are reported as mean ± standard deviation (SD) for continuous variables or as percentages for categorical variables. Student’s *t*-test and the Mann–Whitney U test were used for continuous data, and Pearson’s chi-square test and Fisher’s exact test were used for categorical data. Comparison of the rate of lung function decline and changes in lung function before and after treatment were performed by unpaired *t-*tests with or without Welch’s correction, as appropriate. All statistical analyses were performed using IBM SPSS, Version 21.0 (IBM Corp., Armonk, NY, USA). A two-tailed *p*-value < 0.05 was considered to indicate statistical significance.

## Results

### Baseline characteristics of the subjects

Of the total of 39 patients, 51% were classified as receiving low-dose treatment. The median treatment period was 29.6 months (29.2 months in the low-dose group vs. 30.0 months in the conventional-dose group, *p* = 0.261), and mean blood sirolimus level was 5.5 ± 2.8 ng/ml (3.5 ± 1.3 ng/ml in the low-dose group vs. 7.7 ± 2.3 ng/ml in the conventional-dose group, *p* < 0.001). In the low-dose group, the mean trough level of sirolimus was maintained below 5 ng/ml during the treatment period (Additional file 1: Figure S1). There were no differences between the low- and conventional-dose groups in age, gender, smoking history, prior treatment, extrapulmonary manifestations, lung function or exercise capacity (Table 1). However, more subjects in the low-dose group had TSC (30.0% vs. 0.0%, *p* = 0.020). Most subjects in the low-dose group maintained low blood trough levels due to adverse events (AEs, 67.5%) or a stable disease course after initial treatment (25.0%) (Additional file 2: Table S1). Among patients with TSC-LAM (*n* = 6), four received low-dose sirolimus due to azotaemia (*n* = 1) and treatment history of the renal procedure due to AML (*n* = 3) and remaining two did due to mucositis.

**Table 1:** Comparison of baseline characteristics between the low-dose and conventional-dose groups

Characteristic	Total	Low-dose	Conventional-dose
Number of patients	39	20	19
Age, years	34.8 ± 8.3	34.0 ± 8.7	35.6 ± 8.1
Female	39 (100.0)	20 (100.0)	19 (100.0)
Smoking			
Never	36 (92.3)	19 (95.0)	17 (89.5)
Former	3 (7.7)	1 (5.0)	2 (10.5)
Current	0 (0.0)	0 (0.0)	0 (0.0)
TSC	6 (15.4)	6 (30.0)*	0 (0.0)
Postmenopausal state	2 (5.1)	1 (5.0)	1 (5.3)
Previous treatment			
Medroxyprogesterone	14 (35.9)	7 (35.0)	7 (36.8)
LHRH	4 (10.3)	1 (5.0)	3 (15.8)
Bilateral oophorectomy	1 (2.6)	1 (5.0)	0 (0.0)
History of extrapulmonary manifestation			
Pneumothorax	24 (61.5)	15 (75.0)	9 (47.4)
Angiomyolipoma	14 (35.9)	9 (45.0)	5 (26.3)
Lymphangioleiomyoma	5 (12.9)	1 (5.0)	4 (21.1)
Chylothorax	3 (7.7)	2 (10.0)	1 (5.3)
Lung function			
FEV_1_, % predicted	67.7 ± 26.4	62.9 ± 27.4	72.3 ± 2.3
FVC, % predicted	86.2 ± 14.9	83.3 ± 13.8	88.9 ± 15.7
DLco, % predicted	51.7 ± 21.8	52.6 ± 23.1	50.9 ± 21.2
FEV_1_/FVC ratio	66.2 ± 20.8	64.1 ± 2 4.1	68.2 ± 17.6
6MWT			
Distance, meter	456.9 ± 103.6	458.4 ± 103.5	455.1 ± 106.9
Initial SaO2, %	97.6 ± 1.7	97.7 ± 1.7	97.6 ± 1.7
Lowest SaO2, %	93.7 ± 4.7	94.5 ± 4.9	92.8 ± 4.5
Sirolimus level, ng/mL	5.5 ± 2.8	3.5 ± 1.3*	7.7 ± 2.3

Data are presented as mean ± SD or number (%)

Abbreviations: *TSC* tuberous sclerosis complex, *LHRH* luteinising hormone-releasing hormone, *FEV*_*1*_ forced expiratory volume in 1 s, *FVC* forced vital capacity, *DLco* diffusing capacity of the lung for carbon monoxide, *6MWT* six-minute walk test, *SaO*_*2*_ oxygen saturation

*P < .05 (compared with conventional-dose group)

### Changes in lung function

**Fig. 2:**
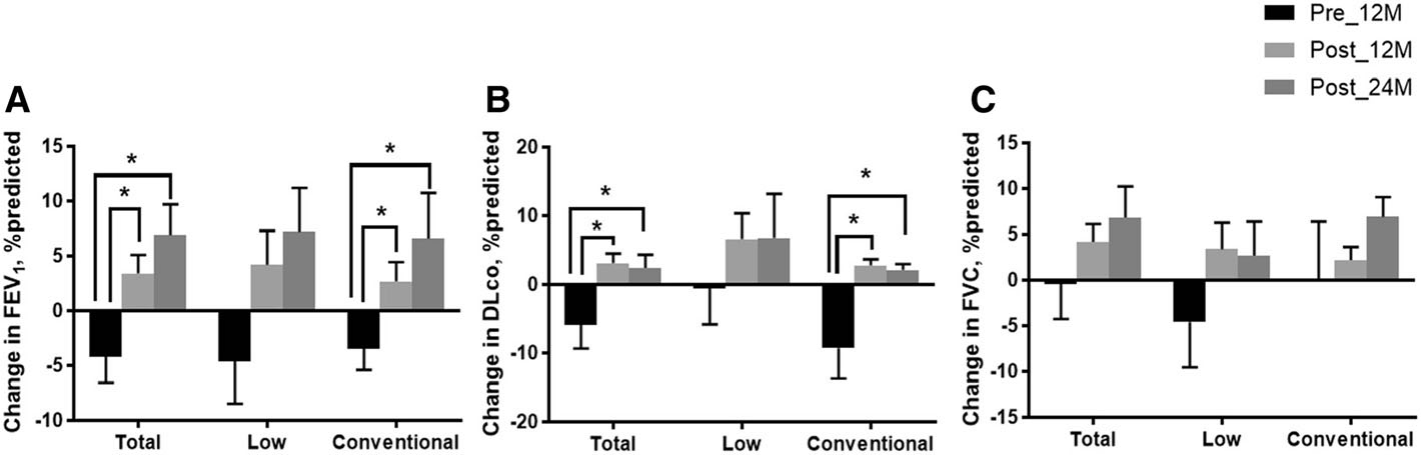
Changes in lung function before and after treatment. Changes in FEV_1_ (**a**), DLco (**b**) and FVC (**c**) before and after treatment. Bars and lines show the mean ± standard error of changes in lung function. Abbreviations: Pre_12M, 12 months before treatment; Post_12M, 12 months after treatment; Post_24M, 24 months after treatment; FEV_1_, forced expiratory volume in 1 s; DLco, diffusing capacity of the lung for carbon monoxide; FVC, forced vital capacity. ^*^p < 0.05

In the whole group, changes in FEV_1_ significantly improved 12 and 24 months after treatment (∆FEV_1_, 3.4 ± 9.3% predicted at 12 months, *p* = 0.004; 6.9 ± 11.5% predicted at 24 months, *p* = 0.007) compared with those before treatment (∆FEV_1_, − 4.2 ± 8.2% predicted; Fig. 2a). The changes in DLco also exhibited similar trends after treatment (∆DLco, 3.1 ± 7.7% predicted at 12 months, *p* = 0.006; 2.4 ± 8.0% predicted at 24 months, *p* = 0.032; Fig. 2b). By contrast, FVC only showed a numerical improvement after treatment (∆FVC_,_ 3.1 ± 7.7% predicted at 12 months, *p* = 0.250; 6.8 ± 14.8% predicted at 24 months, *p* = 0.582; Fig. 2c).

In the low-dose group, FEV_1_ showed an improving trend at 12 and 24 months after treatment (∆FEV_1_, 4.2 ± 11.6% predicted at 12 months, *p* = 0.169; 7.2 ± 12.0% predicted at 24 months, *p* = 0.212) without statistical significance (Fig. 2a). DLco (∆DLco, 6.6 ± 14.0% predicted at 12 months, *p* = 0.145; 6.6 ± 14.0% predicted at 24 months, *p* = 0.250) and FVC (∆FVC_,_ 3.4 ± 10.7% predicted at 12 months, *p* = 0.283; 2.7 ± 11.1% predicted at 24 months, *p* = 0.891) also showed similar trends (Fig. 2b and c). On the other hands, the conventional-dose group showed significant improvements in FEV_1_ (∆FEV_1,_ 2.7 ± 7.0% predicted at 12 months, *p* = 0.010; 6.6 ± 11.7% predicted at 24 months, *p* = 0.015; Fig. 2a) and DLco (∆DLco_,_ 2.8 ± 3.7% predicted at 12 months, *p* = 0.001; 2.1 ± 2.6% predicted at 24 months, *p* = 0.010; Fig. 2b) at 12 and 24 months after treatment; however, only numerical improvements in FVC were observed after treatment (∆FVC, 2.2 ± 5.9% predicted at 12 months, *p* = 0.608; 6.9 ± 6.5% predicted at 24 months, *p* = 0.233; Fig. 2c). There were no differences between the two groups in changes in lung function (FEV_1_, FVC and DLco) before and after treatment.

### Rate of decline in lung function

In the whole group, the rate of decline in FEV_1_ was significantly reduced after treatment (− 0.12 ± 0.47% predicted/month [before] vs. 0.24 ± 0.48% predicted/month [after], *p* = 0.027) compared with before treatment (Additional file 3: Table S2). The rate of decline in DLco (− 0.33 ± 0.61% predicted/month [before] vs. 0.03 ± 0.26% predicted/month [after], *p* = 0.006) was also reduced after treatment, but that of FVC was not.

In the low-dose group, the rate of decline in FEV_1_ showed an improving trend after treatment (− 0.08 ± 0.38% predicted/month [before] vs. 0.19 ± 0.51% predicted/month [after], *p* = 0.264) without statistical significance (Additional file 3: Table S2). There were similar trends in the rates of decline in FVC and DLco after treatment. However, in the conventional-dose group, the rates of decline in FEV_1_ (− 0.26 ± 0.54% predicted/month vs. 0.22 ± 0.38% predicted/month, *p* = 0.024) and DLco (− 0.55 ± 0.58% predicted/month vs. 0.04 ± 0.25% predicted/month, *p* = 0.002) were significantly reduced after treatment (Additional file 3: Table S2).

### Disease progression

**Fig. 3:**
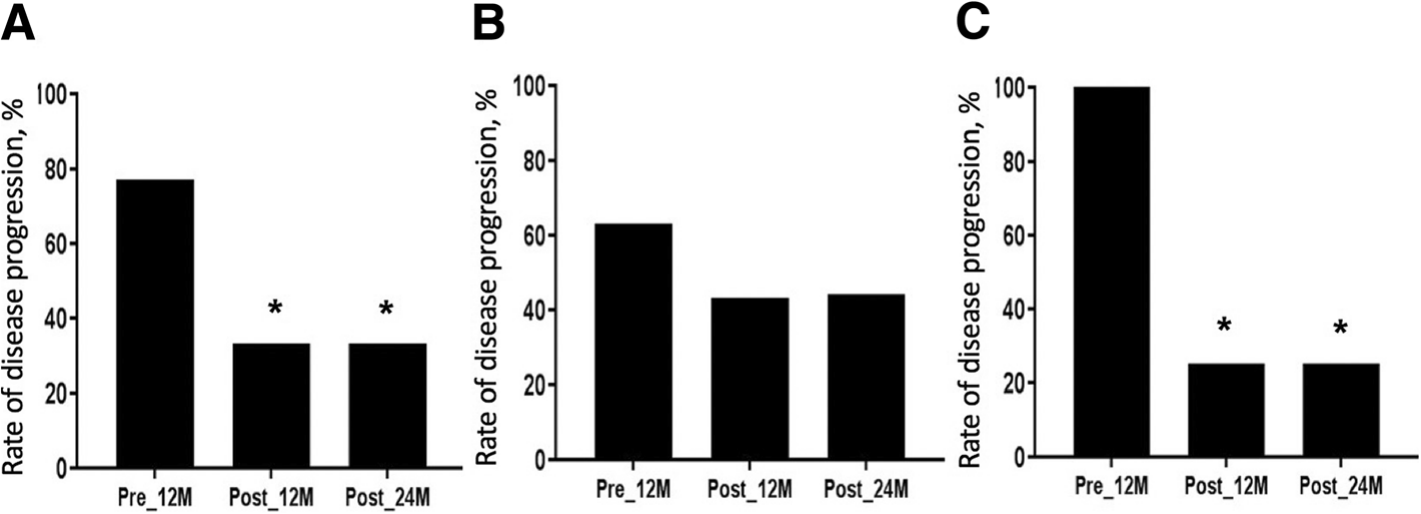
Categorical changes in lung function before and after treatment. Changes in disease progression (any decline in FEV_1_) before and after treatment in all subjects **a**, in the low-dose group **b** and in the conventional-dose group **c**. Bars show the proportion of patients in each group. Abbreviations: Pre_12M, 12 months before treatment; Post_12M, 12 months after treatment; Post_24M, 24 months after treatment; FEV_1_, forced expiratory volume in 1 s. ^*^p < 0.05 (compared with 12 months before treatment)

In the whole group, the rate of disease progression, which was defined as any decline in FEV_1_, decreased after treatment (77% before vs. 33% at 12 months [*p* = 0.008] vs. 35% at 24 months [*p* = 0.024]) (Fig. 3a). In the low-dose group, the rate of disease progression showed a trend towards decrease after treatment (63% before vs. 43% at 12 months [*p* = 0.659] vs. 44% at 24 months [*p* = 0.637]) (Fig. 3b). In the conventional-dose group, however, the rate of disease progression decreased significantly after treatment (100% before vs. 25% at 12 months [*p* = 0.006] vs. 25% at 24 months [*p* = 0.021]) (Fig. 3c).

### Treatment response of extra-pulmonary manifestations

Among 18 patients with extra-pulmonary manifestations, 11 (61.1%) were assessed for treatment responses. The median observation time from the initiation of sirolimus to the last CT follow-up during treatment was 2.9 years (range: 1.4–5.8 years; 2.8 [low] vs. 3.1 [conventional] years, *p* = 0.631). In the whole group, five (45.5%) patients showed improvement and 6 (54.5%) showed stability. Comparison of the results between the low and conventional groups were similar; in the low-dose group, improvement and stability were observed in 2 (28.6%) and 5 patients (71.4%), respectively and in the conventional-dose group, those were observed in 3 (75.0%) and 1 patients (16.7%), respectively (*p* = 0.242).

### Adverse events

Of all patients, 89.7% experienced AEs, averaging 3.46 AEs per patient (Table 2 and Additional file 4: Table S3). The most common AE was hypercholesterolaemia (43.6%), followed by stomatitis (35.9%). The rate of AEs in the low-dose group did not differ from that in the conventional-dose group (85.0% vs. 94.7%, *p* = 0.605). Although there were no significant differences in AEs between the groups, the most common AE in the low-dose group was stomatitis (50.0%), whereas hypercholesterolaemia was the most common in the conventional-dose group (52.6%). The rate of AEs per patient was also comparable in the two groups (3.70 events per patient in the low-dose group and 3.21 events per patient in the conventional-dose group, *p* = 0.406) (Additional file 4: Table S3).

**Table 2:** Comparison of adverse events and serious adverse events between the low-dose and conventional-dose groups

Type of event	Total	Low-dose	Conventional-dose
Number of patients	39	20	19
Adverse events	35 (89.7)	17 (85.0)	18 (94.7)
Hypercholesterolaemia	17 (43.6)	7 (35.0)	10 (52.6)
Stomatitis	14 (35.9)	10 (50.0)*	4 (21.1)
URI	9 (23.0)	3 (15.0)	6 (31.6)
Diarrhoea	7 (17.9)	4 (20.0)	3 (15.8)
Headache	5 (12.8)	4 (20.0)	1 (5.3)
Vaginal bleeding	5 (12.8)	1 (5.0)	4 (21.1)
Acneiform lesions	4 (10.3)	3 (15.0)	1 (5.3)
UTI	4 (10.3)	2 (10.0)	2 (10.5)
Serious adverse events	7 (17.9)	3 (15.0)	4 (21.1)
Infection^a^	4 (10.3)	3 (15.0)	1 (5.3)
Pneumothorax	3 (7.7)	1 (5.0)	2 (10.5)
Malignancy^b^	1 (2.3)	0 (0.0)	1 (5.3)

Data are presented as number (%)

Abbreviations: *URI* upper respiratory infection, *UTI* urinary tract infection

*P < .1 (compared with conventional-dose group)

^a^Infection included pneumonia (*n* = 3) and cellulitis (*n* = 1)

^b^Thyroid cancer

Serious adverse events (SAEs) occurred in 17.9% of all subjects, and there was no significant difference in the rate of SAEs between the low- and conventional-dose groups (15.0% vs. 21.0%, *p* = 0.695) (Table 2). Although there were no significant differences in the rate of AEs between the groups, the most common SAEs were infection (15.0%) in the low-dose group and pneumothorax (10.5%) in the conventional-dose group. There were no deaths during follow-up.

### Discontinuation of treatment

Seven patients (17.9%) permanently discontinued treatment due to planned pregnancy (7.7%), AEs (5.1%), or stable disease status (1.1%). Although the overall discontinuation rate was lower in the low dose group (5.0% [low] vs. 31.6% [conventional], *p* = 0.044) than in the conventional dose group, the discontinuation rate due to AEs were not different between two groups (5.0% vs. 5.3%, *p* = 1.000; Additional file 5: Table S4). Of the two patients who discontinued sirolimus due to AEs, one in the low-dose group discontinued because of stomatitis and one in the conventional-dose group discontinued due to stomatitis and urticaria.

## Discussion

In our current study, low-dose sirolimus may stabilise lung function decline in patients with LAM. The rates of decline in lung function and disease progression showed a trend towards improvement after treatment, similar to the conventional-dose group. The frequency of AEs and the rate of treatment discontinuation due to AEs were also similar in the both low and conventional-dose groups.

Ando et al. showed that low-dose sirolimus, achieving trough levels < 5 ng/mL, effectively resolved chylous effusion in seven patients with LAM and improved lung function (FVC, − 101.0 [before] vs. + 190.0 mL/year [after], *p* = 0.046; FEV_1,_ − 115.4 vs. + 127.8 mL/year, *p* = 0.015) in nine LAM patients without chylous effusion [13]. Our findings also showed trends in improvement of FEV_1_ and disease progression after sirolimus treatment in the low-dose group, although they were not statistically significant. This is consistent with the results of recent studies [20–22]. Bee et al. demonstrated that lower serum sirolimus level was associated with fewer AEs but not with lower efficacy in FEV_1_ decline in a prospective LAM national cohort [20]. Another mTOR inhibitor, everolimus, has recently been used for treatment of LAM in open-label studies; this agent could also stabilise lung function and reduce AML and lymphangioleiomyomas with tolerable safety when administered in low doses [21, 22].

Although low-dose sirolimus may be cost-effective and capable of reducing AEs during chronic suppressive treatment, this treatment has been thought to be at the expense of inferior efficacy compared with conventional-dose therapy [23]. Our results suggesting the efficacy of low dose sirolimus are consistent with those of previous report [13], but the improvement in lung function was greater in the conventional-dose group than in the low-dose group. Although we did not observe significant improvement in lung function changes after treatment in the low-dose sirolimus group, the rates of disease progression and lung function decline were improved. The absence of statistical significance in the results from the low-dose group might be due to the small number of subjects or the lower efficacy compared with the conventional-dose group.

The proportion of patients with TSC-LAM was greater in the low-dose group than in the conventional-dose group. These findings might be attributed from frequent renal involvement in patients with TSC-LAM, such as AML, renal cyst and renal-cell carcinoma. Because of renal complications, these patients tend to undergo nephrectomy or embolisation more frequently [24]. In our cohort, four of six patients with TSC-LAM underwent renal procedures including embolisation and nephrectomy. Because sirolimus has the potential to exacerbate pre-existing or newly occurring renal lesions by causing massive proteinuria, glomerulonephritis or thrombotic microangiopathy [25], patients with TSC who already have impaired renal function might prefer low-dose sirolimus therapy. Actually, most patients with TSC-LAM in our cohort received low-dose sirolimus due to a potential risk of renal impairment and showed the stable course of lung function changes without discontinuation after treatment.

In the present study, the rates of infection, including upper respiratory infection (23.0% vs. 44.0%), urinary tract infection (10.3% vs. 16.0%) and cellulitis (2.5% vs. 12.0%), were lower than those in a study by Bissler et al. [9]. In particular, the incidence of pneumonia requiring hospitalisation in our total cohort was also lower than that in another study (7.7% vs. 30.0%) [26]. Although sirolimus did not increase the risk of infection compared with the placebo in phase 3 clinical trial involving patients with LAM [10], considering the increased risk of infection with sirolimus treatment in transplant patients [27, 28], these results were probably due to the effect of low-dose sirolimus. The rates of stomatitis (35.9% vs. 68.0%) and diarrhoea (17.9% vs. 28.0%) were also lower in our study than in a conventional-dose study [9]; however, the rate of hypercholesterolaemia in our total subjects was similar to that in patients receiving conventional doses of sirolimus in other studies (43.6% vs. 42.1–52.0%) [9, 29].

The AE rates were comparable between the two treatment groups in our study, although the rate of stomatitis showed a trend towards being higher in the low-dose group. However, it should be noted that this was a retrospective study, and most patients who were included in the low-dose group had maintained low doses due to AEs, including mostly stomatitis. Nevertheless, the majority of patients with AEs continued sirolimus with conservative therapy, resulting in comparable AE-related discontinuation rates between the two groups (5% [low dose] vs. 5.3% [conventional dose], *p* = 1.000). These findings indicate that the AEs in the low-dose group were not severe enough to cause discontinuation of treatment. Thus, low-dose sirolimus can be considered tolerable for patients who experience AEs while taking conventional doses.

Our study has some important limitations. First, the number of patients included was relatively small, especially for analysis of efficacy in each group. This is related to the lack of statistical significance in subgroup analysis. However, our results showed that the reduction in lung function before treatment was changed to an increase in lung function after treatment in both groups. Second, this was a retrospective study conducted in a single tertiary referral centre; however, the demographic features and lung function of our patients were comparable to those in other studies. Finally, the proportion of patients with TSC-LAM was higher in the low-dose group than in the conventional-dose group. Nevertheless, baseline lung function did not differ between the two groups, and a recent study showed that there were no differences in changes of FEV_1_, DLco and cyst scores between patients with TSC-LAM and those with sporadic LAM [30]. Therefore, the difference in the proportion of patients with TSC-LAM between two groups would not affect the results of lung function analysis.

## Conclusions

In conclusion, our data suggest that low-dose sirolimus may stabilise lung function decline in patients with LAM who have moderately impaired lung function, and that low-dose sirolimus may be less effective than conventional-dose therapy in preventing lung function decline and disease progression. Based on our results, low-dose sirolimus could be considered a treatment option in patients with LAM, especially those who suffer from intolerable AEs or who have a long-term stable course. Further prospective studies are warranted to confirm these findings.

## Additional files

Additional file 1: **Figure S1.** The mean blood trough level of sirolimus during the first two years of treatment. Dots and error bars show the mean ± 95% confidential interval (DOCX 13 kb)

Additional file 2: **Table S1.** Reasons to maintain low-dose sirolimus treatment. (DOCX 18 kb)

Additional file 3: **Table S2.** Comparison of the rates of decline in lung function between the low-dose and conventional-dose groups. (DOCX 19 kb)

Additional file 4: **Table S3.** Comparison of adverse events and serious adverse events between the low-dose and conventional-dose groups. (DOCX 19 kb)

Additional file 5: **Table S4.** Comparison of reasons for treatment discontinuation between the low-dose and conventional-dose groups (DOCX 13 kb)

## Abbreviations

AEs: Adverse events
AML: Angiomyolipoma
DLco: The diffusing capacity of the lung for carbon monoxide
FEV_1_: Forced expiratory volume in 1 s
FVC: Forced vital capacity
LAM: Lymphangioleiomyomatosis
mTORC1: Mammalian target of the rapamycin complex 1
SAEs: Serious adverse events
SD: Standard deviation
TSC: Tuberous sclerosis complex

## References

[1] Henske EP, McCormack FX (2012). Lymphangioleiomyomatosis - a wolf in sheep's clothing. J Clin Invest.

[2] Harari S, Torre O, Cassandro R, Moss J (2015). The changing face of a rare disease: lymphangioleiomyomatosis. Eur Respir J.

[3] Harari S, Torre O, Moss J (2011). Lymphangioleiomyomatosis: what do we know and what are we looking for?. Eur Respir Rev.

[4] McCormack FX (2008). Lymphangioleiomyomatosis: a clinical update. Chest.

[5] Ballou LM, Lin RZ (2008). Rapamycin and mTOR kinase inhibitors. J Chem Biol.

[6] Kenerson HL, Aicher LD, True LD, Yeung RS (2002). Activated mammalian target of rapamycin pathway in the pathogenesis of tuberous sclerosis complex renal tumors. Cancer Res.

[7] Lee L, Sudentas P, Donohue B, Asrican K, Worku A, Walker V (2005). Efficacy of a rapamycin analog (CCI-779) and IFN-gamma in tuberous sclerosis mouse models. Genes, chromosomes & cancer.

[8] Goncharova EA, Goncharov DA, Li H, Pimtong W, Lu S, Khavin I (2011). mTORC2 is required for proliferation and survival of TSC2-null cells. Mol Cell Biol.

[9] Bissler JJ, McCormack FX, Young LR, Elwing JM, Chuck G, Leonard JM (2008). Sirolimus for angiomyolipoma in tuberous sclerosis complex or lymphangioleiomyomatosis. N Engl J Med.

[10] McCormack FX, Inoue Y, Moss J, Singer LG, Strange C, Nakata K (2011). Efficacy and safety of sirolimus in lymphangioleiomyomatosis. N Engl J Med.

[11] Davies DM, de Vries PJ, Johnson SR, McCartney DL, Cox JA, Serra AL (2011). Sirolimus therapy for angiomyolipoma in tuberous sclerosis and sporadic lymphangioleiomyomatosis: a phase 2 trial. Clin. Cancer Res.

[12] Branco F, Cavadas V, Osorio L, Carvalho F, Martins L, Dias L (2011). The incidence of cancer and potential role of sirolimus immunosuppression conversion on mortality among a single-center renal transplantation cohort of 1,816 patients. Transplant Proc.

[13] Ando K, Kurihara M, Kataoka H, Ueyama M, Togo S, Sato T (2013). The efficacy and safety of low-dose sirolimus for treatment of lymphangioleiomyomatosis. Respir Investig.

[14] Gupta N, Finlay GA, Kotloff RM, Strange C, Wilson KC, Young LR, Taveira-DaSilva AM, Johnson SR, Cottin V, Sahn SA (2017). Lymphangioleiomyomatosis diagnosis and management: high-resolution chest computed tomography, Transbronchial lung biopsy, and pleural disease management. An official American Thoracic Society/Japanese respiratory society clinical practice guideline. Am J Respir Crit Care Med.

[15] Wanger J, Clausen J, Coates A, Pedersen O, Brusasco V, Burgos F, Casaburi R, Crapo R, Enright P, Van Der Grinten C (2005). Standardisation of the measurement of lung volumes. Eur Respir J.

[16] Miller MR, Hankinson J, Brusasco V, Burgos F, Casaburi R, Coates A, Crapo R, Enright P, van der Grinten CP, Gustafsson P (2005). Standardisation of spirometry. Eur Respir J.

[17] Macintyre N, Crapo RO, Viegi G, Johnson DC, van der Grinten CP, Brusasco V, Burgos F, Casaburi R, Coates A, Enright P (2005). Standardisation of the single-breath determination of carbon monoxide uptake in the lung. Eur Respir J.

[18] ATS Committee on Proficiency Standards for Clinical Pulmonary Function Laboratories (2002). ATS statement: guidelines for the six-minute walk test. Am J Respir Crit Care Med.

[19] Therasse P, Arbuck SG, Eisenhauer EA, Wanders J, Kaplan RS, Rubinstein L (2000). New guidelines to evaluate the response to treatment in solid tumors. European Organization for Research and Treatment of Cancer, National Cancer Institute of the United States, National Cancer Institute of Canada. J Natl Cancer Inst.

[20] Bee J, Fuller S, Miller S, Johnson SR (2018). Lung function response and side effects to rapamycin for lymphangioleiomyomatosis: a prospective national cohort study. Thorax.

[21] Avdeev S, Makarova M, Merzhoeva Z, Chuchalin A (2014). Treatment with low-dose everolimus in patients with sporadic lymphangioleiomyomatosis (LAM). Eur Respir J.

[22] Mohammadieh AM, Bowler SD, Silverstone EJ, Glanville AR, Yates DH (2013). Everolimus treatment of abdominal lymphangioleiomyoma in five women with sporadic lymphangioleiomyomatosis. Med J Aust.

[23] McCormack FX (2014). Chronic sirolimus therapy for lymphangioleiomyomatosis. Am J Respir Crit Care Med.

[24] O'Callaghan FJ, Noakes MJ, Martyn CN, Osborne JP (2004). An epidemiological study of renal pathology in tuberous sclerosis complex. BJU Int.

[25] Marti HP, Frey FJ (2005). Nephrotoxicity of rapamycin: an emerging problem in clinical medicine. Nephrol Dial Transplant.

[26] Neurohr C, Hoffmann AL, Huppmann P, Herrera VA, Ihle F, Leuschner S, von Wulffen W, Meis T, Baezner C, Leuchte H (2011). Is sirolimus a therapeutic option for patients with progressive pulmonary lymphangioleiomyomatosis?. Respir Res.

[27] Fisher A, Seguel JM, de la Torre AN, Wilson D, Merchant A, Arora RK, Koneru B (2004). Effect of sirolimus on infection incidence in liver transplant recipients. Liver Transpl.

[28] Alsuwaidi AR, George JA, Almarzooqi S, Hartwig SM, Varga SM, Souid A-K (2017). Sirolimus alters lung pathology and viral load following influenza a virus infection. Respir Res.

[29] Taveira-DaSilva AM, Hathaway O, Stylianou M, Moss J (2011). Changes in lung function and chylous effusions in patients with lymphangioleiomyomatosis treated with sirolimus. Ann Intern Med.

[30] Taveira-DaSilva AM, Jones AM, Julien-Williams P, Yao J, Stylianou M, Moss J (2015). Severity and outcome of cystic lung disease in women with tuberous sclerosis complex. Eur Respir J.

