# Supplementary material for: Efficacy and safety of low-dose Sirolimus in Lymphangioleiomyomatosis
Source: Orphanet J Rare Dis. 2018 Nov 14;13:204. doi: 10.1186/s13023-018-0946-8 (PMC6236936; doi:10.1186/s13023-018-0946-8)
Supplement: Supplementary file 1 — Figure S1. The mean blood trough level of sirolimus during the first two years of treatment. Dots and error bars show the mean ± 95% confidential interval (DOCX 13 kb) [file 13023_2018_946_MOESM1_ESM.docx]

**Figure S1. The mean blood trough level of sirolimus during the first two years of treatment**

Dots and error bars show the mean ± 95% confidential interval
