# Supplementary material for: Efficacy and safety of low-dose Sirolimus in Lymphangioleiomyomatosis
Source: Orphanet J Rare Dis. 2018 Nov 14;13:204. doi: 10.1186/s13023-018-0946-8 (PMC6236936; doi:10.1186/s13023-018-0946-8)
Supplement: Supplementary file 2 — Table S1. Reasons to maintain low-dose sirolimus treatment. (DOCX 18 kb) [file 13023_2018_946_MOESM2_ESM.docx]

**Table S1. Reasons to maintain low-dose sirolimus treatment**

| **Reason** | N (%) |
| --- | --- |
| Adverse event | 13 (67.5) |
| Mucositis | 8 (37.5) |
| Diarrhoea | 2 (5.0) |
| Leukopenia | 2 (5.0) |
| Underlying azotaemia | 1 (2.5) |
| Stable disease after initial treatment | 6 (25.0) |
| Poor compliance due to concerns about AEs | 1 (2.5) |

Data are presented as number (%).

AEs**,** adverse events
