# Supplementary material for: Efficacy and safety of low-dose Sirolimus in Lymphangioleiomyomatosis
Source: Orphanet J Rare Dis. 2018 Nov 14;13:204. doi: 10.1186/s13023-018-0946-8 (PMC6236936; doi:10.1186/s13023-018-0946-8)
Supplement: Supplementary file 3 — Table S2. Comparison of the rates of decline in lung function between the low-dose and conventional-dose groups. (DOCX 19 kb) [file 13023_2018_946_MOESM3_ESM.docx]

**Table S2. Comparison of the rates of decline in lung function between the low-dose and conventional-dose groups**

| Measurement | Group | Pretreatment | Posttreatment | p-value |
| --- | --- | --- | --- | --- |
| FEV_1_, % predicted/month | Total | −0.12 ± 0.47 | 0.24 ± 0.48 | 0.027 |
|  | Low-dose | −0.08 ± 0.38 | 0.19 ± 0.51 | 0.264 |
|  | Conventional-dose | −0.26 ± 0.54 | 0.22 ± 0.38 | 0.024 |
| FVC, % predicted/month | Total | −0.01 ± 0.88 | 0.23 ± 0.44 | 0.319 |
|  | Low-dose | 0.16 ± 0.86 | 0.29 ± 0.59 | 0.679 |
|  | Conventional-dose | −0.05 ± 0.93 | 0.18 ± 0.26 | 0.563 |
| DLco, % predicted/month | Total | −0.33 ± 0.61 | 0.03 ± 0.26 | 0.006 |
|  | Low-dose | −0.13 ± 0.62 | 0.02 ± 0.28 | 0.679 |
|  | Conventional-dose | −0.55 ± 0.58 | 0.04 ± 0.25 | 0.002 |

Data are presented as mean ± standard deviation.

FEV_1_, forced expiratory volume in 1 second; FVC, forced vital capacity; DLco, diffusing capacity of the lung for carbon monoxide.
