# Supplementary material for: Efficacy and safety of low-dose Sirolimus in Lymphangioleiomyomatosis
Source: Orphanet J Rare Dis. 2018 Nov 14;13:204. doi: 10.1186/s13023-018-0946-8 (PMC6236936; doi:10.1186/s13023-018-0946-8)
Supplement: Supplementary file 4 — Table S3. Comparison of adverse events and serious adverse events between the low-dose and conventional-dose groups. (DOCX 19 kb) [file 13023_2018_946_MOESM4_ESM.docx]

**Table S3. Comparison of adverse events and serious adverse events between the low-dose and conventional-dose groups**

| Type of event | No. of events per patient | | |
| --- | --- | --- | --- |
|  | Total | Low-dose | Conventional-dose |
| Adverse events | 3.46 | 3.70 | 3.21 |
| Hypercholesterolaemia | 0.82 | 0.70 | 1.00 |
| Stomatitis | 0.56 | 0.90* | 0.21 |
| URI | 0.49 | 0.50 | 0.47 |
| Diarrhoea | 0.18 | 0.20 | 0.16 |
| Headache | 0.21 | 0.30 | 0.11 |
| Vaginal bleeding | 0.15 | 0.10 | 0.21 |
| Acneiform lesions | 0.13 | 0.20 | 0.05 |
| UTI | 0.10 | 0.10 | 0.11 |
| Serious adverse events | 0.18 | 0.15 | 0.21 |
| Infection^¶^ | 0.10 | 0.15 | 0.05 |
| Pneumothorax | 0.08 | 0.05 | 0.10 |
| Malignancy† | 0.02 | 0.00 | 0.05 |

Data are presented as mean values. Abbreviations: URI, upper respiratory infection; UTI, urinary tract infection.

**P <.05* (compared with conventional-dose group)

^¶^Infection included pneumonia (n = 3) and cellulitis at the removal site of a pigtail catheter (n = 1).

^†^Thyroid cancer.
