# Supplementary material for: Efficacy and safety of low-dose Sirolimus in Lymphangioleiomyomatosis
Source: Orphanet J Rare Dis. 2018 Nov 14;13:204. doi: 10.1186/s13023-018-0946-8 (PMC6236936; doi:10.1186/s13023-018-0946-8)
Supplement: Supplementary file 5 — Table S4. Comparison of reasons for treatment discontinuation between the low-dose and conventional-dose groups (DOCX 13 kb) [file 13023_2018_946_MOESM5_ESM.docx]

**Table S4. Comparison of reasons for treatment discontinuation between the low-dose and conventional-dose groups**

| Reason | Total | Low-dose | Conventional-dose |
| --- | --- | --- | --- |
| Number of patients | 7 (17.9) | 1 (5.0)* | 6 (31.6) |
| Planned pregnancy | 3 (7.7) | 0 (0.0) | 3 (15.8) |
| Adverse event | 2 (5.1) | 1 (5.0) | 1 (5.3) |
| Stable disease | 1 (2.6) | 0 (0.0) | 1 (5.3) |

Data are presented as number (%).

**P <.05* (compared with conventional-dose group)
